# Multiple Manifold Clustering Using Curvature Constrained Path

**DOI:** 10.1371/journal.pone.0137986

**Published:** 2015-09-16

**Authors:** Amir Babaeian, Alireza Bayestehtashk, Mojtaba Bandarabadi

**Affiliations:** 1 Department of Mathematics, University of California San Diego, San Diego, California, United States of America; 2 Department of Computer Science, Oregon Health and Science University, Portland, Oregon, United States of America; 3 Department of Informatics Engineering, University of Coimbra, Coimbra, Portugal; Hong Kong University of Science and Technology, HONG KONG

## Abstract

The problem of multiple surface clustering is a challenging task, particularly when the surfaces intersect. Available methods such as Isomap fail to capture the true shape of the surface near by the intersection and result in incorrect clustering. The Isomap algorithm uses shortest path between points. The main draw back of the shortest path algorithm is due to the lack of curvature constrained where causes to have a path between points on different surfaces. In this paper we tackle this problem by imposing a curvature constraint to the shortest path algorithm used in Isomap. The algorithm chooses several landmark nodes at random and then checks whether there is a curvature constrained path between each landmark node and every other node in the neighborhood graph. We build a binary feature vector for each point where each entry represents the connectivity of that point to a particular landmark. Then the binary feature vectors could be used as a input of conventional clustering algorithm such as hierarchical clustering. We apply our method to simulated and some real datasets and show, it performs comparably to the best methods such as K-manifold and spectral multi-manifold clustering.

## Introduction

**Fig 1:**
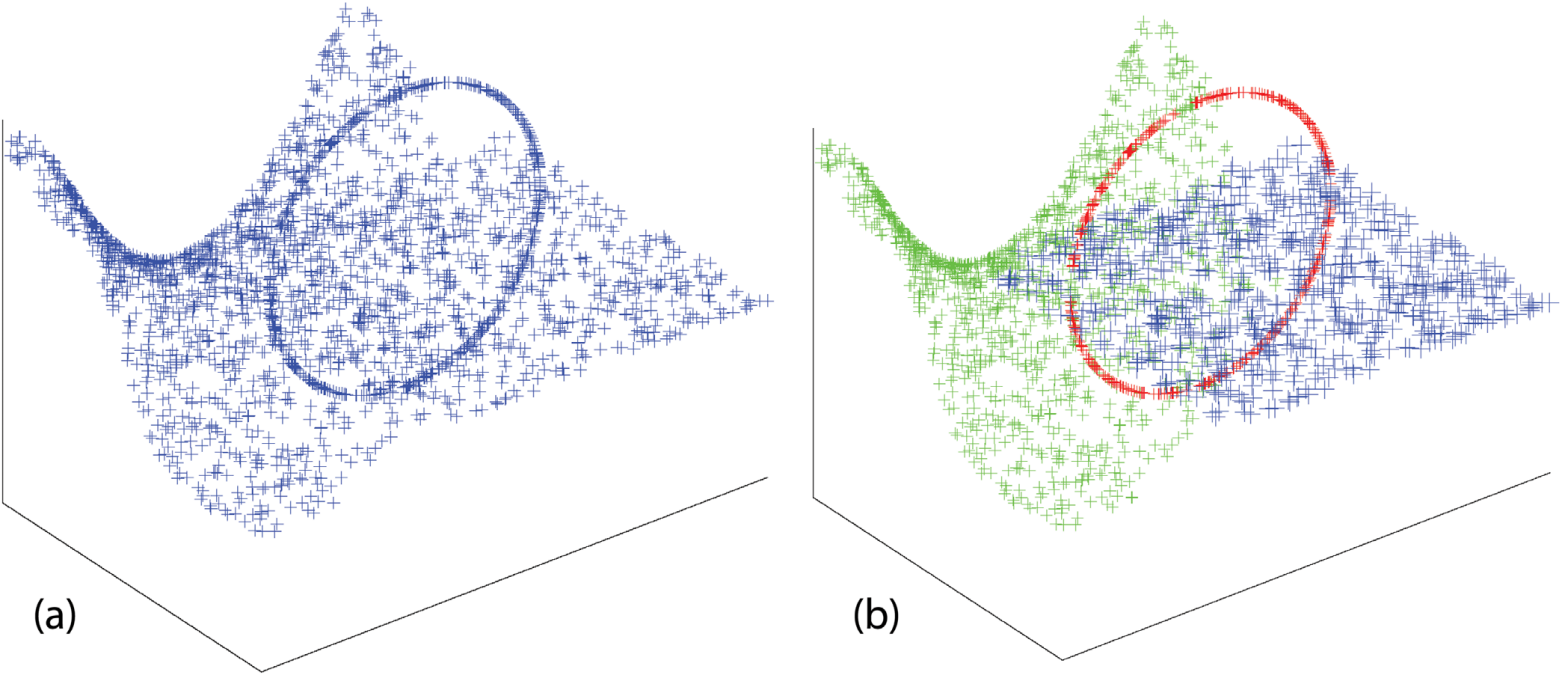
Simulated data illustrating the problem of multi-manifold clustering. Left: 3D data. Right: output from our method.

We consider the problem of clustering points that are sampled in the vicinity of multiple surfaces embedded in Euclidean space, with a particular interest in the case where these intersect. The goal is multi-manifold clustering, which amounts to labeling each point according to the surface it comes from. This stylized problem may be relevant in a number of applications, such as the extraction of galaxy clusters [1] and road tracking [2] and target tracking [3–7] after some preprocessing. In motion segmentation [8–10] and in face recognition [11–13], the underlying surfaces are usually assumed to be affine or, more generally, algebraic. Here we focus on a nonparametric setting where the main assumption is that the surfaces are smooth—see Fig 1 for an example. This appears to be necessary to remove ambiguities in the problem of separating intersecting surfaces.

Several approaches have been proposed in this context. Most methods are designed for the case where the surfaces do not intersect [14–16], while others work only when the surfaces that intersect have different intrinsic dimension or density [17, 18]. The method of [19] is only able to separate intersecting curves. Methods that purposefully aim at resolving intersections are fewer. Souvenir et al. [20] implement some variant of K-means [21–23] where the centers are surfaces. Guo et al. [24] propose to minimize a (combinatorial) energy that includes local orientation information, using a tabu search. The state-of-the-art method lies in methods based on local principal component analysis (PCA). An early proposal was the elaborate multiscale spectral method of [25], while the clustering routine of [26]—developed in the context of semi-supervised learning—inspired the works of [27] and [28].

We propose a markedly different approach based on connecting points to landmarks via curvature-constrained paths. It can be seen as a constrained variant of [29]. Isomap was specifically designed for dimensionality reduction in the single-manifold setting, and in particular, cannot handle intersections. It has been used in different applications [30–34]. The curvature constraint on paths is there to prevent connecting points from one cluster to points from a different, intersecting cluster. The resulting algorithm is implemented as a simple variation of Dijkstra’s algorithm. Our method is simpler than the previous proposals in the literature and performs comparably to the best methods, both on simulated and real datasets.

The rest of the paper is organized as follows. In the next section we explain the notion of curvature constrained shortest-path and it’s connection with the curvature constrained shortest-path. In the algorithm section we present our algorithm for multi-manifold clustering and compare it with three currently applied methods and give a theoretical guarantee for that. In the numerical experiments section we performed multiple numerical experiments on simulated and real data. Robustness of method to noise and choice of constraint is discussed as well. In the discussion section we discuss and outline our future work and development of our algorithm.

## Constrained path

### Neighborhood graph

Neighborhood graphs play a central role in manifold learning, exploiting the fact that smooth submanifolds are locally flat. Recall that a neighborhood graph is a graph with vertices the sample points *x*_1_, …, *x*_*N*_. We consider two types of neighborhood structure [35]:

- **ɛ*-ball.* *x*_*i*_ and *x*_*j*_ are connected if ‖*x*_*i*_ − *x*_*j*_‖ ≤ *ɛ*, where ‖ ⋅ ‖ denotes the Euclidean norm.
- **k*-nearest neighbor*. *x*_*i*_ and *x*_*j*_ are connected if *x*_*j*_ is among the *k*-nearest neighbors of *x*_*i*_ (in the Euclidean metric), or vice-versa.

### Angle constraint

The central idea in this paper is the use of *constrained* shortest-path distances in a neighborhood graph. The paths are constrained in order to control their smoothness. The constrained shortest-path distances are used to estimate geodesic distances reliably, even when the surface self-intersects, thus allowing us to mimic Isomap. We use the fact that the constrained and unconstrained shortest-path distances are similar for points belonging to the same submanifold, while usually different for points belonging to different submanifolds.

For an ordered triplet of points (*x*, *y*, *z*) in ℝ^*D*^, define its angle as

$$∠\left(x,y,z\right)=∠\left(xy,yz\right)=\cos^{-1}(\frac{\left\langle y-x,z-y\right\rangle }{∥y-x∥∥z-y∥})\in [0,\pi ]$$ (1)

We say that a sequence of points (*x*_*i*_1__, …, *x*_*i*_*m*__) is *θ*-angle constrained if the angles between successive segments are all bounded by *θ*, meaning

$$∠(x_{i_{t-1}},x_{i_{t}},x_{i_{t+1}})\leq \theta ,\;\forall t=2,\cdots ,m-1.$$ (2)

### Curvature constraint

For an ordered triplet of points (*x*, *y*, *z*) in ℝ^*D*^, we define the curvature as

$$\mathrm{curv}\left(x,y,z\right)=\{\begin{array}{cc} {\left(R\left(x,y,z\right)\right)}^{-1}, & \text{if}\;∠\left(x,y,z\right)<\frac{\pi }{2},\\ \infty , & \text{otherwise}, \end{array}$$ (3)

where ∠ stands for the angle and *R*(*x*, *y*, *z*) is the radius of the circle passing through *x*, *y*, *z*.

$$R\left(x,y,z\right)=\frac{\sqrt{{∥x-y∥}^{2}+{∥z-y∥}^{2}+2∥x-y∥∥z-y∥\cos∠(x,y,z)}}{\sin∠(x,y,z)}.$$ (4)

with *R*(*x*, *y*, *z*) = ∞ if *x*, *y*, *z* are aligned.

**Definition**. For a curvature *κ* > 0, we say that a path (*x*_*i*_1__, …, *x*_*i*_*m*__) is *κ*-constrained if $\mathrm{curv}(x_{i_{t-1}},x_{i_{t}},x_{i_{t+1}})\leq \kappa ,\;\forall t=2,\ldots ,m-1$.

**Definition**. For every point *y* in the graph the annulus neighborhood of that point is a set of points on the graph that are within *ɛ*/2 and *ɛ* distance of point *y*.

**Fig 2:**
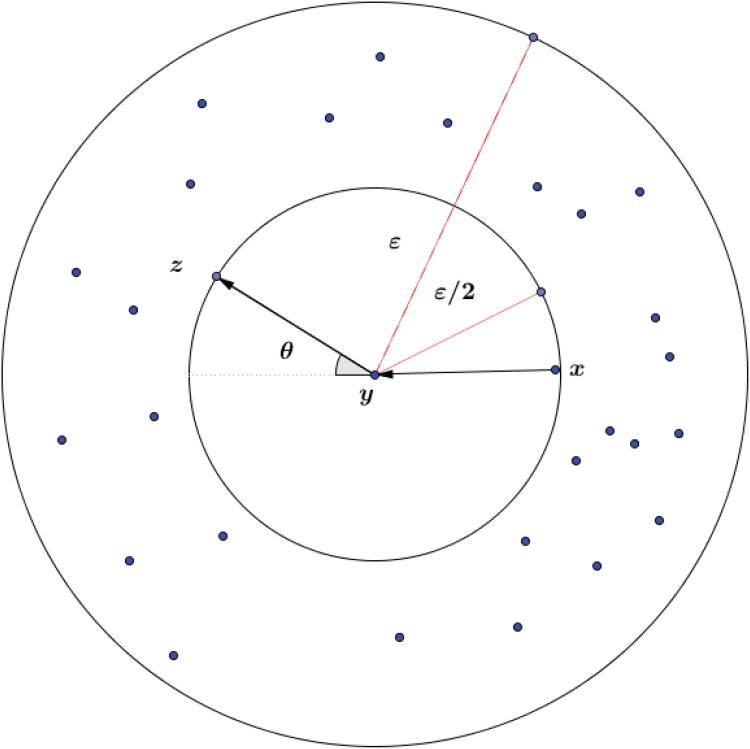
*x* and *z* lie in annulus neighborhood of point *y*.

Fig 2 shows three D-dimensional points *x*, *y*, *z* which form vertices of a triangle such that *x* and *z* belong to the annulus neighborhood of point *y*. Under above assumption the angle constraint ∠(*x*, *y*, *z*) < *θ* where *θ* < *π*/2 implies curvature constraint curv(x,y,z)<κ where $\kappa =2sin\left(\theta \right)/\sqrt{\frac{\varepsilon^{2}}{2}\left(1+cos\left(\theta \right)\right)}$.

### Our algorithm

To compute these constrained shortest-path distances we use a simple modification of Dijkstra’s algorithm. Then input of the algorithm is the *KNN* graph, angle or curvature constraint and landmark points. Algorithm compute the constrained shortest path distance between every land mark points and all the points in the graph by taking in to the account that if the path between three consecutive points violate the constraint then that path would be considered as infinity or actually there is no path. The main difference of our algorithm with Dijkstra’s algorithm is that we need to check the constraint during Dijkstra’s process when we consider the neighboring nodes of our current node. Suppose we are in current node *x* and *y* and *z* are the only neighbors of *x* and these two nodes in Dijkstra’s process are not visited yet and the weight from *x* to *y* is less than weight of *x* to *z*, But the path from *parnts*[*x*], *x*, *y* violate the constraint but *parnt*[*x*], *x*, *z* does not violate, so in this case we consider *parent*[*x*], *x*, *z* as the right path. There is no guarantee always there exist such a path, which means in this case there is no path from that land mark and a given point in the graph. See algorithm 1 below. When applied to a neighborhood graph with maximum degree Δ, its computational complexity is *O*(Δ*N* log*N*) per source point.

**Algorithm 1** Curvature Constrained Shortest Path Algorithm

**Input**: neighborhood graph 𝒢 including weights of all the connected vertecies, the landmark *x*_ℓ_ where ℓ = 1, …, *L*, angle or curvature constraint *θ*.

**for** ℓ = 1 **to** *L* **do**

*ss* = ℓ, *t* = ℓ, *m* = 0.

 For each vertex *i* of the graph(*i* = 1, …, *N*): *distance*[*i*] = *Inf*, *cost*[*i*] = *Inf*, *parent*[*i*] = *Inf*, *temporary*[*i*] = 0.

 At the beginning for each vertex *i* of the graph, there is no path from that vertex to the landmark *x*_ℓ_, i.e., *path*[ℓ][*i*] = [].

**for** *j* = 1 **to** *N* **do**

  Update *distance*[*i*] for each *i* ∈ *neighbors*(*t*) by the weight of edge between the vertex *i* and *t*.

**for** *i* = 1 **to** *N* **do**

**if** *distance*[*i*] + *m* < *cost*[*i*] **then**

**if** curv(*i*, *t*, *ss*) < κ or *t* == *ss* **then**

     Update *parent*[*i*] = *t* and *cost*[*i*] = *distance*[*i*] + *m*.

**end if**

**end if**

**end for**

  Compute *temporary* + *cost* vector then find the minimum element of these vector as well as the vertex *I* with minimum element.

  Update *m*, i.e., *m* = *min*(*temporary* + *cost*).

**if** *parent*[*i*] ∼ = *Inf* **then**

   Update *path*[ℓ][*I*] by appending vertex *I* to the end of *path*[ℓ][*parent*[*I*]].

**end if**

*temporary*[*I*] = *Inf*.

*distance*[*i*] = *Inf* for all *i* = 1, …, *N*.

  Update *t* = *I* and choose *ss* as the parent of t, i.e., *ss* = *parent*[*t*].

  Update weights of edge from vertex *ss* to *t* and from vertex *ss* to *t* by *Inf* in order to avoid revisiting vertices. Our graph is a directed graph, so it is possible to have edges in both directions between two vertices.

**end for**

**end for**

**Output**: Constrained shortest-Path from each vertex *i* of the graph and each landmark ℓ.

## Multi-Manifold Clustering

### Existing methods

The last decade saw a flurry of propositions aiming at clustering data points when the underlying clusters are not convex, and in particular, in the situation where the points are sampled near low-dimensional objects. We gave a few references in the Introduction and now want to elaborate on three of them, [20, 36] and [27], as we will use them as benchmarks in our experiments. Our choice was dictated by performance, code availability and relevance to our particular setting.

The method of [25] renders impressive results but is hard to tune, having many parameters, while the method of [28] is very similar to that of [27] and the code was not publicly available at the moment of writing this paper. The other methods for multi-manifold clustering that we know of were not designed to resolve intersections of clusters of possibly identical intrinsic dimensions and sampling densities.

We chose the subspace clustering method of [36] among a few others methods that perform well in this context.

#### K-Manifolds

Souvenir et al. [20] suggest an algorithm that mimics K-means, replacing centroid points with centroid submanifolds. The method starts like Isomap by building a neighborhood graph and computing shortest path distances within the graph. After randomly initializing a *K*-by-*n* weight matrix *W* = (*w*_*ki*_), where *w*_*ki*_ represent the probability that point *i* belongs to the *k*th cluster, it alternates between an M-Step and an E-Step. In the M-Step, for each *k*, the points are embedded in ℝ^*K*^ using a weighted variant of multidimensional scaling using the weights (*w*_*ki*_ : *i* = 1, …, *n*). In the E-Step, for each *k* and *i*, the normal distance of point *x*_*i*_ to the cluster *k* is estimated as

$$\delta_{ki}=\frac{\sum_{j}w_{kj}\left(d\left(x_{i},x_{j}\right)-d_{k}\left(x_{i},x_{j}\right)\right)}{\sum_{j}w_{kj}},$$

where *d*(*x*_*i*_, *x*_*j*_) denotes the shortest path distance in the neighborhood graph and *d*_*k*_(*x*_*i*_, *x*_*j*_) denotes the Euclidean distance in the *k*th embedding, between points *x*_*i*_ and *x*_*j*_. The weights are then updated as $w_{ki}\propto \exp\left(-d_{ki}^{2}/\sigma^{2}\right)$ such that ∑_*k*_ *w*_*ki*_ = 1 for all *i*, where *σ* ^2^ is chosen automatically.

#### Spectral Curvature Clustering

Chen et al. [36] proposed a spectral method for subspace clustering—the setting where the underlying surfaces are affine. We will compare our method to theirs when the surfaces are affine, and also when the surfaces are curved. The latter is done as a proof of concept, for it will be very clear that it cannot handle curved surfaces, like any other method for subspace clustering we know of. The procedure assumes that all subspaces are of same dimension *d*, which is a parameter of the method. For each (*d*+2)-tuple, *x*_*i*_1__, …, *x*_*i*_*d*+2__, it computes a notion of curvature *C*_*i*_1_, …, *i*_*d*+2__ which measure how well approximated this (*d* + 2)-tuple is by an affine subspace of dimension *d*. After reducing the tensor **C** = (*C*_*i*_1_, …, *i*_*d*+2__ : *i*_*t*_ = 1, …, *N*) spectral graph partitioning [15] is applied.

#### Spectral Multi-Manifold Clustering

Wang et al. [27] suggest a spectral method using a dissimilarity that factors in the Euclidean distance and the discrepancy between the local orientation of the data. The surfaces are assumed to be of same dimension *d* known to the user. First, a mixture of probabilistic principal component analyzers [37] are fitted to the data, approximating the point cloud by a union of *d*-planes. This is used to estimate the tangent subspace at each data point. The dissimilarity between two points is then an increasing function of their Euclidean distance and the principal angles between their respective affine subspaces. These dissimilarities are fed to the spectral graph partitioning method of [15].

### Our algorithm

We consider the following problem of surface clustering. Given a sample *x*_1_, …, *x*_*n*_ ∈ ℝ^*D*^ sampled from 𝒮_1_ ∪ ⋯ ∪ 𝒮_*K*_, where for each *k*, 𝒮_*k*_ is a smooth, but possibly self-intersecting surface, label each point according to the surface it belongs to. Our algorithm is quite distinct from all the other methods for multi-manifold clustering we are aware of, although it starts by building a *q*-nearest neighbor graph like many others. The idea is very simple and amounts to clustering together points that are connected by an angle-constrained path in the neighborhood graph. Take two surfaces *S*_1_ and *S*_2_ intersecting at a strictly positive angle. Then for ‘most’ pairs of data points *x*_*i*_1__ ∈ *S*_1_ and *x*_*i*_2__ ∈ *S*_2_, a path in the graph going from *x*_*i*_1__ to *x*_*i*_2__ has at least one large angle between two successive edges, on the order of the incidence angle between the surfaces; while for ‘most’ pairs of data points *x*_*i*_1__, *x*_*i*_2__ ∈ *S*_1_, there is a path with all angles between successive edges relatively small. To speedup the implementation, we select *M* landmarks (with *M* slightly larger than *K*) at random among the data points and only identify what data points are connected to what landmark via a *κ*-constrained path in the graph. *M* and *κ* are parameters of the algorithm. Let *ξ*_ℓ*i*_ = 1 if point *i* and landmark ℓ are connected that way, and *ξ*_ℓ*i*_ = 0 if not. We use *ξ*_*i*_: = (*ξ*_ℓ*i*_ : ℓ = 1, …, *M*) as feature vectors that we group together and cluster using hierarchical clustering with complete linkage.

**Algorithm 2** Path-Based Clustering (PBC)

**Input**: data (*x*_*i*_); parameters *q*, *K*, *M*, *κ*

Build *q*-nearest neighbor graph

Choose *M* landmarks are random

**for** *i* = 1 **to** *n* **do**

 For each landmark ${\hat{x}}_{\ell }$, identify which points *x*_*i*_ it is connected to via a *κ*-constrained path in the graph, and set *ξ*_ℓ*i*_ = 1 if so, and *ξ*_ℓ*i*_ = 0 otherwise.

**end for**

Group and then apply hierarchical clustering to the feature vectors *ξ*_1_, …, *ξ*_*n*_ to find *K* clusters, where *ξ*_*i*_: = (*ξ*_ℓ*i*_ : ℓ = 1, …, *M*).

#### Intersections

We are most interested in the case where the surfaces intersect. Concretely, given *K* compact, simply connected submanifolds *S*_1_, …, *S*_*K*_ ⊂ ℝ^*D*^ of maximum pointwise curvature bounded by *κ* < ∞, we consider the noisy mixture distribution

$$x=s+z,\;s∼\sum_{k=1}^{K}\pi_{k}\mu_{S_{k}}\;z∼\mu_{B(0,\tau )},$$ (5)

where *μ*_*S*_ denotes the uniform distribution over set *S*.

**Definition**. We say that two smooth submanifolds *S*_1_ and *S*_2_ have incidence angle *α* ∈ (0, *π*/2) at an intersection point *x* ∈ *S*_1_∩*S*_2_ if the smallest nonzero principal angle between the tangent subspaces of *S*_1_ and *S*_2_ at *x* is equal to *α*.

We assume that, for any pair of underlying surfaces *S*_*k*_ and *S*_ℓ_, the minimum incidence angle between them at any point along their intersection is at least *α* > 0. The compactness of the surfaces imply the existence of a function *ω*(*ɛ*) → 0 with *ɛ* → 0 such that, for any *ɛ* > 0, if dist (*x*, *S_k_*) ∨ dist (*x*, *S_ℓ_*) ≤ ε, then dist (*x*, *S_k_* ∩ *S_ℓ_*) ≤ ω (ε). Otherwise, there is *C* > 0 such that, for all *m* ≥ 1, there is *x*_*t*_ satisfying dist (*x_t_, S_k_*) ∨ dist (*x_t_, S_ℓ_*) ≤ 1/*m* and dist (*x_t_, S_k_* ∩ *S_ℓ_*) ≥ *C*. By the fact that (*x*_*t*_) is bounded (since *S*_*k*_ and *S*_ℓ_ are), there is a subsequence that converges to some *x*, which is necessarily in both *S*_*k*_ and *S*_ℓ_ since these sets are closed. At the same time, dist (*x*, *S_k_* ∩ *S_ℓ_*) ≥ *C* by continuity of the distance function, which is a contradiction. In fact, the assumption on the incidence angle implies that *ω*(*ɛ*) ≤ *Cɛ* for some *C* > 0 not depending on *ɛ*—but this is much longer to prove.

#### Landmarks

A key ingredient to the success of the procedure is that there is at least one landmark chosen from each cluster that is far away from any other cluster. That said, we work with the stronger condition that *all* chosen landmarks are more than *ɛ* away from the other clusters, which leads to simplifications latter on in the discussion.

**Theorem 1** $p_{M}^{*}\left(\varepsilon +2\tau \right)\to 1$, *the probability that all M landmarks are away from the other clusters by at least ɛ converges to 1*.

*Proof*. The probability of selecting a point from Eq (5) with *s* ∈ *S*_*k*_ away from any other cluster by at least *ɛ* is $p_{k}\left(\varepsilon \right):=\pi_{k}(1-\mu_{S_{k}}\left({⋃}_{\ell \neq k}S_{\ell }^{\varepsilon }\right))$, where *S* ^*ɛ*^ denotes the points in ℝ^*D*^ within distance *ɛ* of *S*. Then the probability that all *M* landmarks are away from the other clusters by at least *ɛ* is equal to

$$p_{M}^{*}\left(\varepsilon +2\tau \right):={\left(\sum_{k}p_{k}\left(\varepsilon +2\tau \right)\right)}^{M}$$ (6)

where we used the triangle inequality. By dominated convergence, when *ɛ*, *τ* → 0, *p_k_*(*ε* + 2*τ*) → *π_k_*(1 - *μ_S_k__* (⋃_*ℓ*≠*k*_ *S_ℓ_*)) = *π_k_*, implying $p_{M}^{*}\left(\varepsilon +2\tau \right)\to 1$, if we assume that *μ*_*S*_*k*__(*S*_ℓ_) = 0 when ℓ ≠ *k*, which is a very mild assumption we make hereafter.

## Numerical Expriments

### Synthetic Data

**Fig 3:**
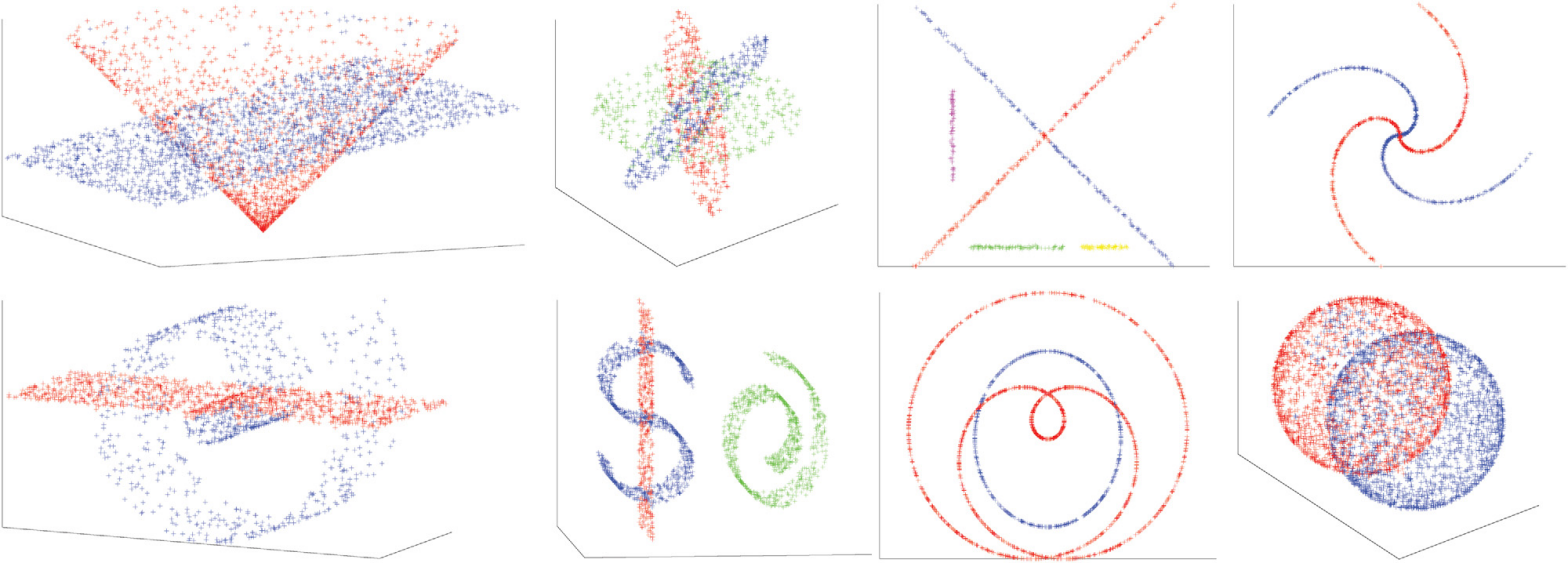
Result of our method on 8 synthetic datasets.

**Fig 4:**
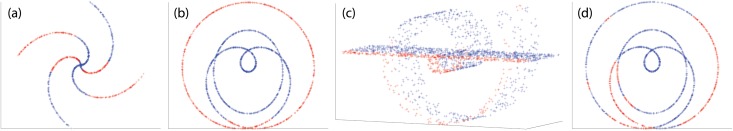
Examples where the other methods fail. (a) SCC, (b) SMMC, (c, d) K-Manifolds.

In our experiments we use annulus neighborhood of points to build our graph. The constraint is angle constraint which is easier to tune. The synthetic datasets we generated are similar to those appearing in the literature. We have applied our method in 8 synthetic data including: Three Planes(TP), Two Spirals (TS1), Five Segments(FS), Dollar-Sign and Plane and Roll(DPR), Roll and Plane(RP), Cone and Plane(CP), Two Spheres(TS2), Rose Curve and Circle(RC). Fig 3 shows the performance of our algorithm on eight synthetic data set. The misclustering rates for our method, and the other three methods, are presented in Table 1, where we see that our method achieves a performance at least comparable to the best of the other three methods on each dataset. To compute the accuracy of clustering we remove a few ambiguous points close by intersection. Spectral Curvature Clustering (SCC) works well on linear manifolds (as expected) while it fails when there is curvature (Fig 4a). K-Manifolds fails in the more complicated examples (Fig 4c and 4d). We found that this algorithm is very slow since it has to compute the shortest path between all the points, so that we could not apply it to some of the largest datasets. We mention that it assumes that clusters intersect, and otherwise does not work properly. Our method and Spectral Multi-Manifolds Clustering (SMMC) perform comparably on most datasets, but SMMC fails in the Rose Curve and Circle example (Fig 4b). Since the path from self-intersecting rose is very smooth at the intersection SMMC cannot capture the correct manifold. The local geometric information of the sampled data can not be used to construct a suitable affinity matrix. Pairwise affinity between two local tangent spaces at the intersection is very similar. For more detail please look at Fig 3 in SMMC paper. Our method can take care of very smooth self-intersecting manifolds or multiple manifolds at the intersection with a choice of an appropriate constraint. We note that K-Manifold, SCC and SMMC all require that all surfaces are of same dimension, which is a parameter of these methods, while our method does not need knowledge of the intrinsic dimensions of the surfaces and can operate even when these are different.

**Table 1:** Clustering accuracy on synthetic data.

Data set	K-Manifolds	SCC	SMMC	PBC-Angle-Annulus	PBC-Curvature
TP	97.1%	97.8%	99.5%	99.6%	99.6%
TS1	95.2%	54.8%	99.7%	99.2%	99.1%
FS	59.1%	94.9%	99.6%	98.1%	98.0%
DPR	50.2%	-	99.6%	99.7%	99.5%
RP	56.5%	-	97.6%	96.7%	96.9%
CP	-	-	99.6%	97.9%	98.1%
TS2	-	-	96.7%	98.6%	98.4%
RCC	62.9%	-	64.8%	99.8%	99.7%

### Clustering of 2D Image Data

**Fig 5:**
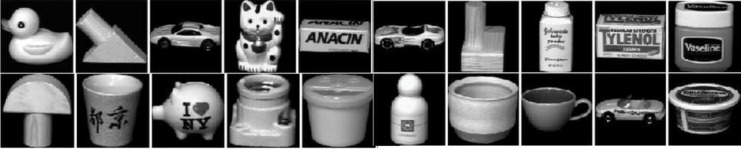
The 20 objects from the COIL-20 database. This figure is similar but not identical to the original image, and is therefore for illustrative purposes only.

In this section we apply our method on the COIL-20 dataset (Fig 5) which includes 1440 gray-scale images of 20 objects. Each object contains 72 images taken by a camera at different angles. The original resolution of each image is 128 × 128. We first projected the dataset onto the top 10 principal components, then apply our path-based clustering algorithm. We tested our method on the three very similar objects 3, 6 and 19. The algorithm is 99% accurate (misclusters only 2 images out of 216) bringing a significant improvement over the state-of-the-art result of 70% reported in [27]. Lastly, we evaluated our method on the whole dataset obtaining an 83.6% accuracy, improving on the 70.7% accuracy reported in [27]. (Here we used the top 20 principal components.) Since in this case we have 20 different classes, we increased the number of landmarks to 100 to make sure we sampled that at least a few landmarks from each class.

### Clustering of Human Motion Sequences

**Fig 6:**
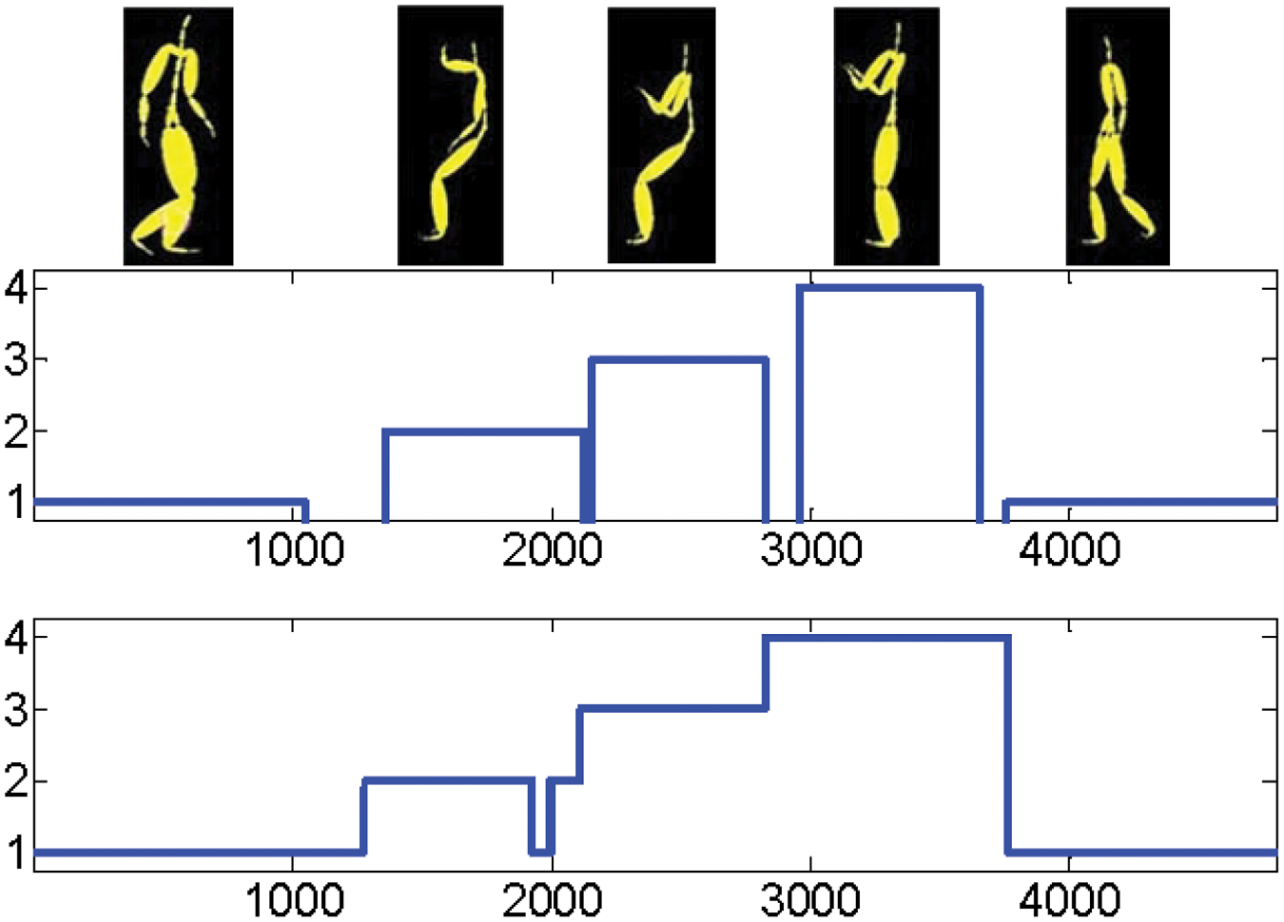
Result of human activity segmentation using Path-Based Clustering. There are 4 activities: walking (1), looking (2), sitting (3) and standing (4). Top: a sample of the sequence. Middle: ground truth. Bottom: output of our algorithm.

In computer vision clustering of human motion sequences into different class of activities performed by a subject is referred to temporal segmentation. In this section we test our algorithm on a sequence of video frames including different activities performed by a subject. We choose 4 mixed actions from subject 86, trial number 9 of the CMU MoCap dataset. This is the data set used by [20].? The data consists in a temporal sequence of 62-dimensional representation of the human body via markers in ℝ^3^. One motion sequence of 4794 frames and corresponding result of path-based multi-manifold clustering are given in Fig 6. Four activities are labeled from 1 to 4. We do not label the frames where the subject switches from one action to another because of the uncertainty about the true activity. In experimental results we used the same data set as K-Manifold used in their paper. You can refer to their paper for more details. In this data SCC fails since it is suitable for linear surfaces with the intersection, it wont work for nonlinear manifolds.

### Segmentation of Video Sequences

In this section we consider the problem of partitioning a video sequence into different scenes. We consider the same video sequence used in [36, 38]. The video is an interview from Fox News containing 135 image frames of size 294 × 413. Firstly we change each RGB image frame to the gray scale intensity image, then resize it to an 74 × 104 image. After concatenating all pixels of each image and putting into a vector of size 7696, we construct a matrix of size 135 × 7696 where each row represents a frame of the original video sequence. Applying our algorithm on this matrix we get a perfect clustering (100%). We repeated the experiment, this time projecting the data onto the top 10 principal components as done in [36, 38], obtaining a matrix of size 135 × 10. We still get a 100% accuracy, for an even wider range of parameters.

## Discussion

### Robustness to Noise

**Fig 7:**
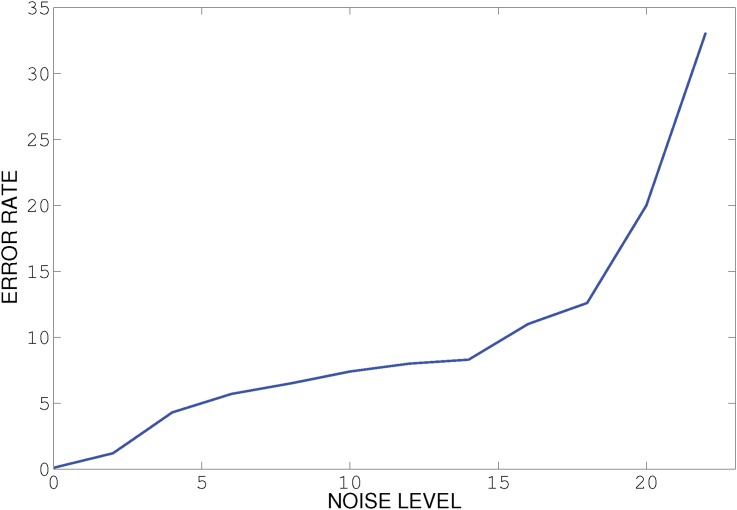
Effect of noise on performance of our algorithm on two intersecting curves shown in Fig 8.

**Fig 8:**
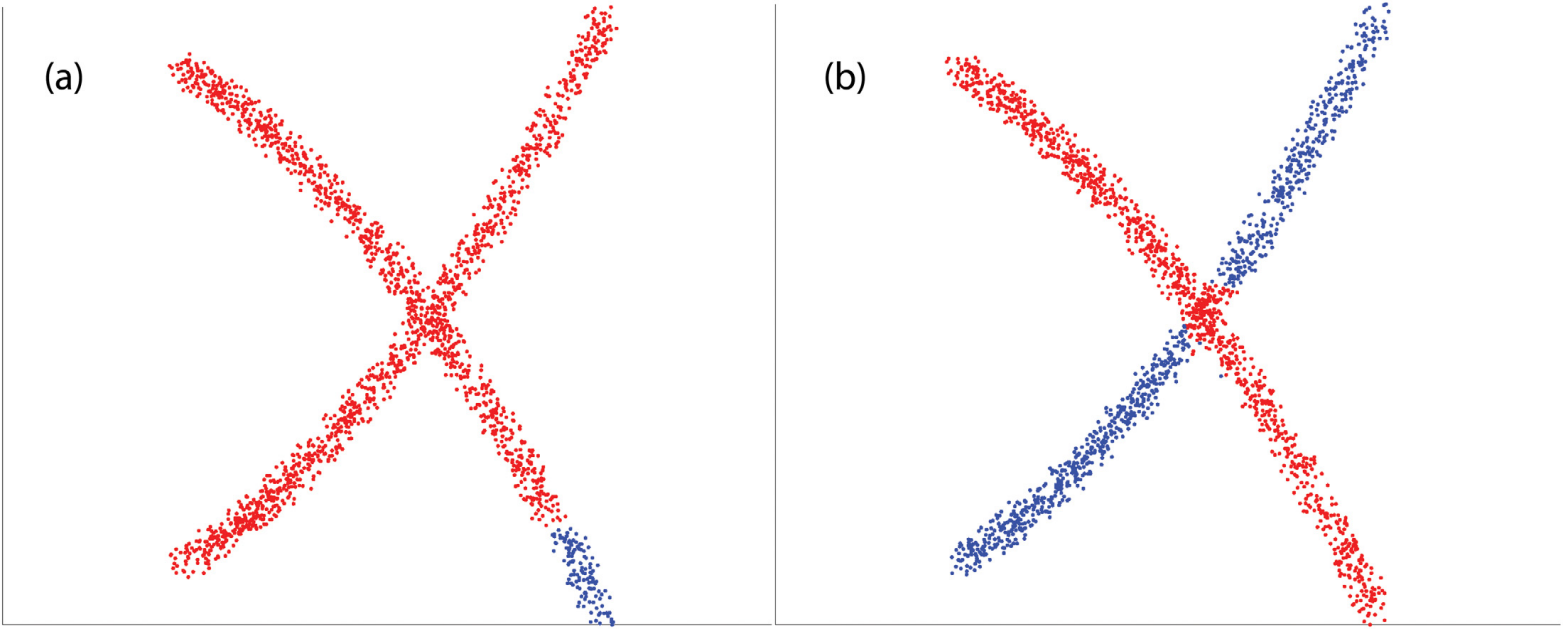
Example of noisy data. (a) output of SMMC. (b) output of PBC.

We note that all the other methods we know of for multi-manifold clustering do not perform well unless the noise level is quite small. As it appears in our method when we increase the amount of noise the possibility of connecting points from different surface with curvature constrained path increases. Fig 7 shows the error rate for two intersecting curves with addition of standard uniform noise, as it can be illustrated, when we increase the noise, notion of two different surface or manifold would be ambiguous where we can say all the points belong to one manifold. All other three methods fail to capture the correct manifold with even small noise where our method still perform well with 20% noise. See Fig 8 for an example with a substantial amount of noise, where SMMC fails while PBC succeeds.

### The choice of constraint

**Fig 9:**
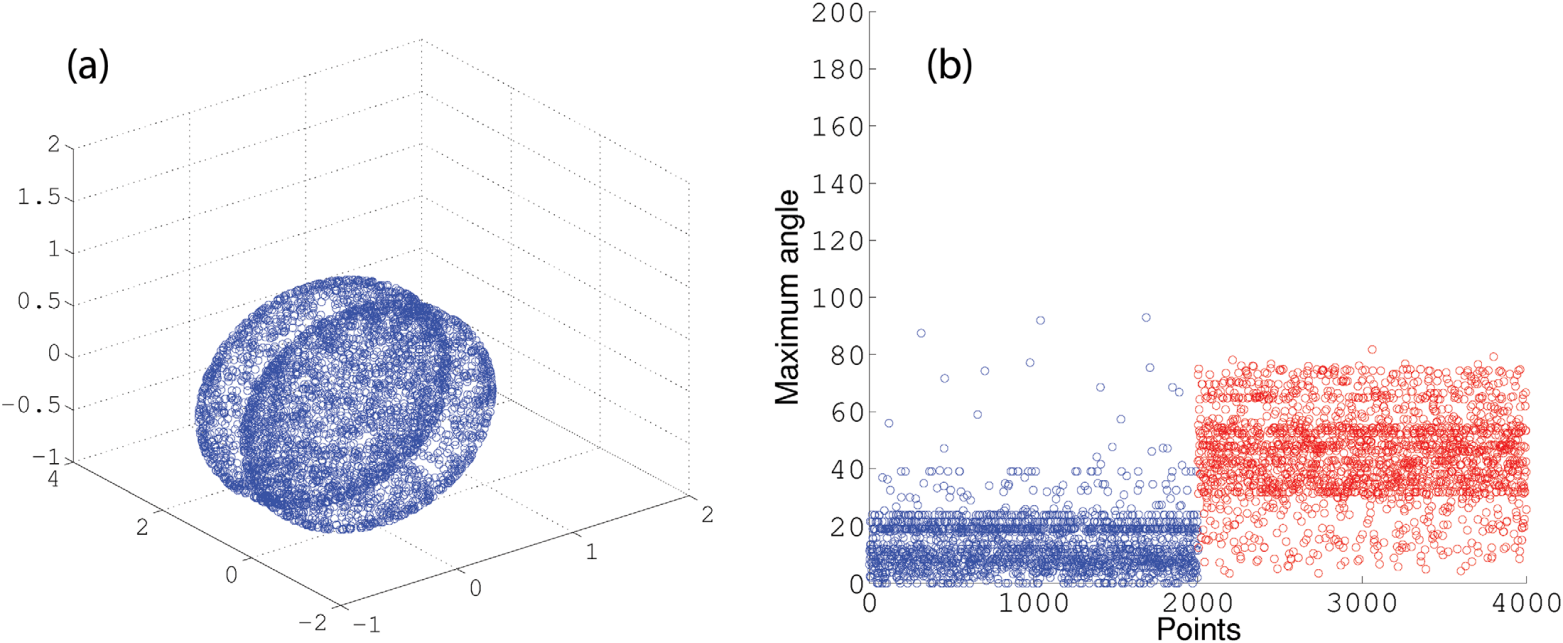
(a) two intersecting Sphere. (b) the distribution of maximum angle in unconstrained shortest-paths between points and a given landmark.

One of the main challenges of our algorithm is the choice of the angle constraint with annulus graph, since we deal with multiple manifolds with intersection. The large angle constraint causes the points from different manifolds to be connected using constrained shortest-path. This ultimately leads to multiple manifolds being clustered as one class. We also considered implementing the small constraint, however, this constraint does not allow us to accurately capture the structure of the manifold. Fig 9 shows two intersecting spheres and the distribution of maximum angle in an unconstrained shortest-path between all the points and a given landmark. The distributions of maximum angle for the points within the same sphere as landmark belongs to (blue) is separable from the distributions of maximum angle for the points within the sphere that landmark does not belong to (red). This illustration guides us to the idea that with the small amount of labeled points we are able to find the appropriate angle constraint. In another experiment we started with an angle constraint of 50° and used 1% of the points in each cluster as labeled data. We then compared the performance of our algorithm on the labeled data. In order to find the optimum angle constraint we increased or decreased our angle constraint by a certain factor. We initially begin with dividing our angle constraint by a factor of 2, until the error ceases to decrease. In the case that the error increases, we increase the angle constraint by a factor of 4/3. In most cases we were able to find the optimal angle constraint within 5 iterations. As it can be understood from Fig 9 the distribution of the maximum angle of the points within a class follows a flat distribution. By having a small number of labeled points we are able to capture the distribution of the maximum angle for the rest of the points in that class.

### Computational Complexity

The algorithm is quite fast. Building a symmetric *q*-nearest neighbor graph using cover trees [39] takes order *O*(*qN* log*N*), where the implicit constant depends exponentially on the intrinsic dimensions of the surfaces and linearly on the ambient dimension *D*. The angle-constrained pathfinder routine is a simple variant of Dijkstra’s algorithm, whose implementation by Fibonacci heaps runs in *O*(*qN* log*N*). Hence, calling this routine once for each landmark costs *O*(*qMN* log*N*). Grouping the feature vectors *O*(*FMN*) and then clustering them by complete linkage costs *O*(*F* ^2^ log*F*), where *F* is the (data-dependent) number of distinct feature vectors *ξ*_*i*_, often of the order of *K* in our experiments.

### Relation to Landmark-Isomap

As [14] argued that Local Linear Embedding [40] could be readily used for clustering non-intersecting manifolds, in a sense, we show that a constrained version of Landmark-Isomap [41] can be used to cluster possibly intersecting manifolds

## Conclusion

In this paper we proposed a new method to cluster multiple manifolds with the intersection which works based on shortest constrained path. We applied our method to synthetic and some real datasets and demonstrated that it performs comparably to the best methods such as K-manifold and spectral multi-manifold clustering. We are currently experimenting with variants—some based on other constraints—that would lead to path-based clustering algorithms that perform at least as well in practice as algorithm 1, and are consistent in the large-sample limit.
